# The Metabolic State of *E. coli* Influences Fosfomycin Efficacy and Promotes Resistance Evolution

**DOI:** 10.1021/acsinfecdis.5c01013

**Published:** 2026-02-10

**Authors:** Andreas Verhülsdonk, Amelie Stadelmann, Fabian Smollich, Johanna Rapp, Daniel Straub, Hannes Link

**Affiliations:** † Interfaculty Institute of Microbiology and Infection Medicine, 9188 University of Tübingen, 72076 Tübingen, Germany; ‡ Cluster of Excellence “Controlling Microbes to Fight Infections”, University of Tübingen, 72076 Tübingen, Germany; § M3 Research Center, University of Tübingen, Otfried-Müller-Str. 37, 72076 Tübingen, Germany; ∥ Quantitative Biology Center (QBiC), University of Tübingen, Otfried-Müller-Str. 37, 72076 Tübingen, Germany

**Keywords:** fosfomycin resistance, CRISPR interference, bacterial metabolism, heteroresistance, peptidoglycan biosynthesis

## Abstract

The phosphonic antibiotic fosfomycin is a bacterial cell wall synthesis inhibitor that targets MurA, the first enzyme in the peptidoglycan pathway. Transporter loss or enzymatic inactivation confers resistance to fosfomycin, but whether the metabolic state of a bacterium influences the efficacy of this antibiotic has not been characterized. Here, we used an *Escherichia coli* CRISPR interference library targeting 1,515 metabolic genes to identify metabolic activities that influence fosfomycin efficacy. We discovered that knockdowns of ATP synthase and pyruvate kinase genes lead to a regrowth phenotype, whereby cells resume growth after an initial phase of killing. By following up on this phenotype with population analysis profile tests and repeated treatment cycles, we found evidence that a heteroresistant population may promote the evolution of fosfomycin resistance. Whole-genome sequencing of the *pykF* CRISPRi strain after 24 h of fosfomycin exposure revealed that the acid stress protein-encoding gene *ibaG*, which is upstream of *murA*, carries a mutation that confers fosfomycin resistance. Metabolome analysis showed accumulation of the MurA substrate phosphoenolpyruvate in regrowing cells, which may compete with fosfomycin for binding to MurA. Transcriptome analysis provided further insight into the mechanism of cell regrowth, including upregulation of genes encoding cell envelope stress response regulators such as *cpxP*. These results suggest that the metabolic state can modulate the efficacy of fosfomycin and contribute to resistance evolution.

## Graphical abstract

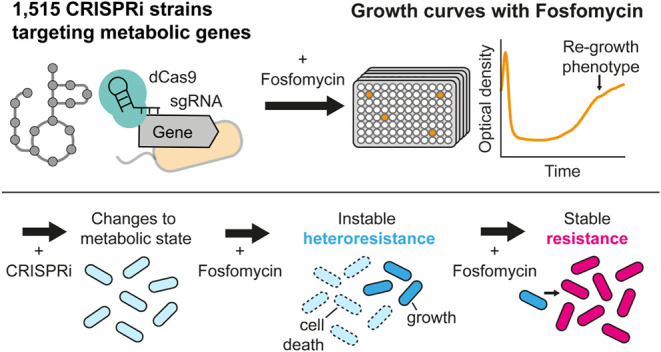

*Escherichia coli* can cause urinary tract infections (UTIs), bloodstream infections, and intra-abdominal infections.^1^ The increasing resistance of *E. coli* to various antibiotics has renewed clinical interest in fosfomycin, which, however, remains on the WHO reserve and watch group.^2^ Fosfomycin is a phosphonic acid-derived antibiotic that inhibits bacterial cell wall synthesis by targeting the enzyme UDP-*N*-acetylglucosamine enolpyruvyl transferase (MurA). MurA catalyzes the first committed step in peptidoglycan synthesis and converts UDP-*N*-acetyl-α-d-glucosamine and phosphoenolpyruvate (PEP) to UDP-*N*-acetyl-α-d-glucosamine-enolpyruvate. Fosfomycin is a structural analogue of PEP and covalently binds at the MurA active site.

Although fosfomycin is highly effective in killing bacterial cells, *E. coli* can evolve resistance against fosfomycin by the loss of function mutations that affect the GlpT and UhpT transporters, which are required for drug uptake, as well as through the acquisition of plasmids encoding fosfomycin-modifying enzymes like the glutathione S-transferase FosA, which enzymatically inactivates the antibiotic.^2–5^ Resistance mutations directly in *murA* have been described,^6^ but they have high fitness costs due to the low mutational flexibility of *murA*.^7^ In addition to such canonical resistance mechanisms, recent studies suggest that bacteria can evade killing of antibiotics like fosfomycin without acquiring resistance mutations.^8^ For example, bacteria can enter a state of *persistence*, which is characterized by the presence of a cell subpopulation that survives antibiotic exposure and recovers after the treatment.^9,10^ A similar phenomenon called *heteroresistance* describes a cell subpopulation that is able to grow in the presence of antibiotics.^9,11,12^ The resulting resistant population can revert to its original sensitive state over the next generations once the antibiotic is removed.^9,11^ Persistence and heteroresistance can contribute to treatment failure and even promote the development of canonical resistance, such as mutations in the drug target.^13,14^

Reduced metabolic activities can also decrease the efficacy of antibiotics.^15^ In case of fosfomycin, mutations in genes that are involved in the synthesis of the regulatory metabolite cyclic AMP (cAMP), like *ptsI* or *cyaA*, can decrease the concentration of cAMP, which in turn decreases the expression of GlpT and UhpT, thus reducing fosfomycin uptake.^3,16^ In *Staphylococcus aureus*, the concentration of PEP is thought to influence the efficacy of fosfomycin,^17^ although evidence for changes in PEP levels is missing. Mutations in UhpA, a direct regulator of the UhpT transporter, also increase fosfomycin survival rates.^18^ Moreover, recent studies have shown that fosfomycin also relies on outer membrane porins OmpF, OmpC, and LamB to enter the cell, and mutations in these genes confer fosfomycin resistance.^4^ Furthermore, phosphonate degradation enzymes and phosphate transporters, identified via high-density transposon mutagenesis, are suspected to modulate the transport systems by affecting intracellular phosphate levels.^19^

Here, we sought to systematically identify metabolic activities that change the susceptibility of *E. coli* to fosfomycin. Therefore, we measured the growth of a metabolism-wide CRISPR interference (CRISPRi) library^20^ during fosfomycin treatment. The library included 1,515 *E. coli* strains, each with a knockdown of a single metabolic gene in the iML1515 metabolic model of *E. coli*.^21^ Using this large-scale functional genomics approach, we identified genes that influence the susceptibility of *E. coli* to fosfomycin. We then followed up on top hits from this screen, which are CRISPRi strains that target ATP synthetase and pyruvate kinases, and found evidence that heteroresistance causes a shift in fosfomycin susceptibility that promotes the evolution of resistance mutations. We identified one of these mutations in *ibaG* ^K45I^, which is located upstream of the fosfomycin target *murA*.

## Results

### CRISPRi Identifies Metabolic Genes That Influence Fosfomycin Efficacy

We screened an *E. coli* CRISPRi library^20^ against fosfomycin, which targets each metabolic gene in the metabolic model iML1515.^21^ The CRISPRi strains have an anhydrotetracycline (aTc)-inducible dCas9 on the genome and a sgRNA on a plasmid, which enables dynamic knockdowns of all metabolism-related genes (Figure 1A). For the screen, we arrayed 1,515 CRISPRi strains on 96-well plates, each targeting one of the 1,515 genes in iML1515.

**Figure 1:**
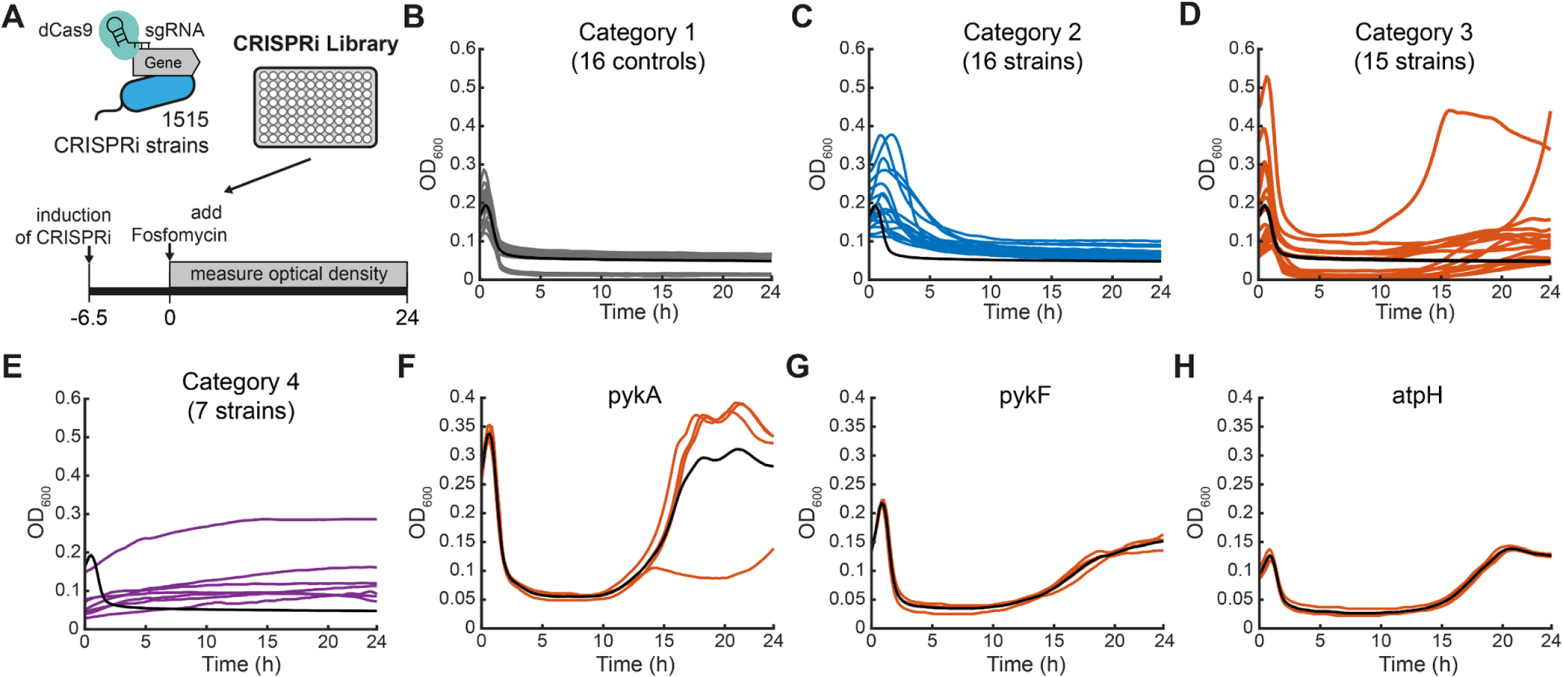
A CRISPRi screen identifies metabolic genes that influence the response of *E. coli*to fosfomycin. (A) Schematic of the CRISPRi library antibiotic screen. The library containing 1,515 CRISPRi strains and controls was induced with aTc for 6.5 h and subsequently cultivated for another 24 h in medium containing 304 μg/mL fosfomycin (*n* = 2). (B) Response of controls (*n* = 16) to the addition of fosfomycin at *t* = 0. (C) Strains in category 2 (16 strains) have an OD decrease phase at least 2 times longer than controls (black line, *n* = 16). (D) Strains in category 3 (15 strains) grew like controls in the first phase (<9 h) and showed OD increases in later phases (>9 h). (E) Strains in category 4 showed increases in OD. (F) Validation of the regrowth phenotype in the *pykA* strain (*n* = 4). The black line is the mean. (G) Same as (F) for the *pykF* strain (*n* = 4). (H) Same as (F) for the *atpH* strain (*n* = 4). Note that the black line in B–E shows the same mean of controls (*n* = 16) as a reference.

First, we used a control strain with a nontargeting sgRNA to determine the minimal inhibitory concentration (MIC) against fosfomycin in liquid broth assays (Figure S1). At a fosfomycin concentration of 76 μg/mL, the control strain showed no growth over a period of 24 h, and we used this concentration of fosfomycin as the MIC in this study. For the CRISPRi screen, we added 304 μg/mL (4× MIC) fosfomycin 6.5 h after induction of dCas9 (Figure 1A). After the addition of fosfomycin, the optical density (OD) of the control strain culture increased for almost 1 h, followed by a decrease in OD, presumably due to cell lysis (Figure 1B). We systematically assigned the strains to four categories by scoring OD time profiles. Category 1 included 1,477 strains that showed a response similar to 16 cultures with the control strain (a CRISPRi strain with a nontargeting sgRNA), suggesting that the knockdown did not affect their susceptibility to fosfomycin. Category 2 consisted of 16 strains with a markedly longer cell lysis phase, indicating that the knockdown influenced the killing activity of fosfomycin (Figure 1C). Category 3 included 15 strains that responded like the control during the first cultivation phase but regrew at later time points (>9 h, Figure 1D). Finally, category 4 consisted of 7 strains that showed no reduction in OD and slowly grew during the 24 h (Figure 1E), thus indicating low-level resistance against fosfomycin.

To confirm the phenotypes of all 38 strains from categories 2 to 4, we repeated the experiment (Table S1). The list of 38 strains for validation was extended to include the pyruvate kinase 1 gene (*pykF*), which showed a small regrowth phenotype in the initial screen but fell below the cutoff (Figure S2A). We also added the remaining 3 ATP synthase genes *atpA*, *atpC,* and *atpF* that showed no (*atpA*) or weak (*atpC*, *atpF*) regrowth in the initial screen (Figure S2B-D). The growth pattern of all strains was confirmed, except the knockdown of *yehY* (encoding the glycine betaine ABC transporter membrane subunit) and *lpxL* (encoding lauroyl acetyltransferase), which grew like the control in all four replicates (Figure S3). The *pykA* and *pykF* strains both showed the regrowth phenotype (Figure 1 F,G). The difference in the intensity of the regrowth phenotype is probably due to the different activity of pyruvate kinases 1 and 2 under different levels of oxygen supply.^22^ All ATP synthase knockdown strains showed the regrowth phenotype, which means that decreasing the abundance of any subunit of the ATP synthase or associated proteins (AtpI) is sufficient for regrowth to occur (Figure 1H, Figure S3).

### Knockdown of Pyruvate Kinase and ATP Synthase Increases MIC

Next, we focused on the killing activity of fosfomycin in the CRISPRi strains and measured colony forming units (CFUs) of the *atpH* strain, *atpB* strain, *pykF* strain, *pykA* strain, and control strain after various times of fosfomycin treatment (Figure 2A). During the first 3 h of the fosfomycin treatment, the killing activity was almost identical in all strains, and CFUs decreased by more than 99.9% (Figure 2A). Killing during this phase is consistent with the results of the CRISPRi screen, where strains with a regrowth phenotype responded similarly to the control strain in the initial phase of treatment. However, after 9 h of treatment, we observed an increase in CFUs for the *pykF*, *atpH*, and *atpB* strains, whereas the *pykA* and control strains showed hardly any CFUs (Figure 2A). Thus, the regrowth phenotype of the *pykF*, *atpH,* and *atpB* strains is characterized by an active increase in cell number, which could be due to a subpopulation that is resistant to fosfomycin (heteroresistance).

**Figure 2:**
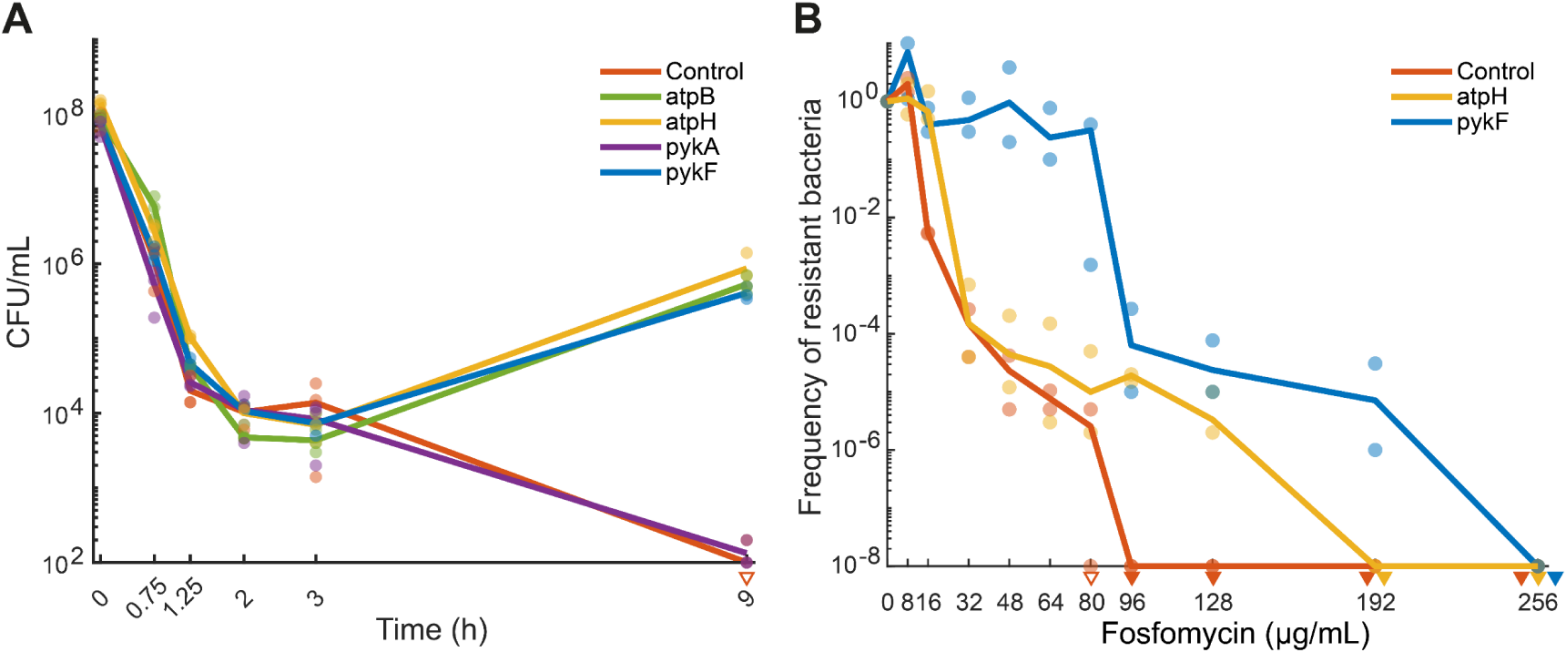
Time-kill assays and population analysis profile tests. In all graphs, empty triangles indicate one replicate below the detection limit, and filled triangles indicate all replicates below detection limits. Before treatment, all strains reached exponential growth at OD > 0.25. (A) Time-kill assay with CRISPRi strains (control, *atpB*, *atpH*, *pykF*, and *pykA*). Strains were incubated for 9 h in minimal glucose medium containing aTc and 304 μg/mL fosfomycin (*n* = 3). Lines indicate a mean of *n* = 3 replicates, and dots represent individual replicates. (B) Population analysis profile (PAP) tests of CRISPRi strains (control, *atpH*, and *pykF*) in minimal glucose medium. Strains were incubated for 24 h on minimal glucose agar plates containing aTc and increasing concentrations of fosfomycin (*n* = 2). Lines indicate a mean of *n* = 2 replicates, and dots represent individual replicates.

We then investigated whether the regrowth phenotype of the *pykF* and *atpH* strains resulted from heteroresistance. Therefore, we performed population analysis profile (PAP) tests^12^ with the two strains and the nontargeting control (Figure 2B). On agar plates with minimal glucose medium, the wild-type strain showed an MIC of 8 μg/mL (Figure 2B). The main population of the *atpH* strain had an MIC of 16 μg/mL, and the *pykF* strain had an MIC of 64 μg/mL, indicating that the knockdowns led to 2-fold and 8-fold MIC increases, respectively. In both strains, a small subpopulation (10^–4^–10^–5^) showed substantially higher MICs compared with their respective main populations. In the *atpH* strain, the MIC increased from 16 μg/mL in the main population to 128 μg/mL in a subpopulation of ca. 1 × 10^–5^ of the cells. This meets the canonical definition of heteroresistance, which requires a resistant subpopulation occurring at a frequency above 10^–7^ and with at least an 8-fold higher MIC than the main population.^12^ In the *pykF* strain, the MIC of a 10^–5^ subpopulation was 192 μg/mL, which is a 3-fold higher MIC than that of the main population. Although this does not meet the definition of heteroresistance, the *pykF* strain showed clear heteroresistance on LB medium, with an MIC of 8 μg/mL for the main population and 256 μg/mL for 10^–4^ of the cells (Figure S4). In contrast, the *atpH* strain behaved similarly to the control strain on the LB medium— both were highly sensitive to fosfomycin but still contained a small fraction of cells with higher MICs (Figure S4).

### CRISPRi Knockdown of *pykF* Promotes the Evolution of a Resistance Mutation in *ibaG*

To determine if the regrowth phenotype is due to stable or unstable heteroresistance, we exposed the control strain, *atpH* strain, and *pykF* strain to repeated cycles of fosfomycin treatment (Figure 3). Therefore, these strains were treated with fosfomycin and recovered after 9 and 24 h to be treated again with fosfomycin for 24 h. The control strain did not recover after 24 h of treatment (Figure 3G). However, 4 out of the 8 replicate cultures of the control strain recovered after 9 h (Figure 3D). In the case of the *pykF* strain, all replicate cultures recovered after 9 h, and they were susceptible to fosfomycin (Figure 3F). However, their regrowth phenotype was more pronounced than that after the initial treatment (Figure 3C), thus indicating that the fraction of heteroresistant bacteria has increased. Importantly, after the initial treatment of 24 h, some replicates grew in the presence of fosfomycin (Figure 3I), which suggests that they had acquired stable resistance mutations. The *atpH* strain showed a similar behavior to the *pykF* strain (Figure 3B,E,H), and 4 of the 9 h treatment cultures showed a slower OD decrease during the first 3 h, which is similar to tolerant strains in category 2 of our initial screen (Figure 1C). In summary, since both strains showed a progressive shift toward resistance, we hypothesized that a fraction of heteroresistant cells may withstand the 4× MIC of fosfomycin and that this promotes the development of stable fosfomycin resistance mutations.

**Figure 3:**
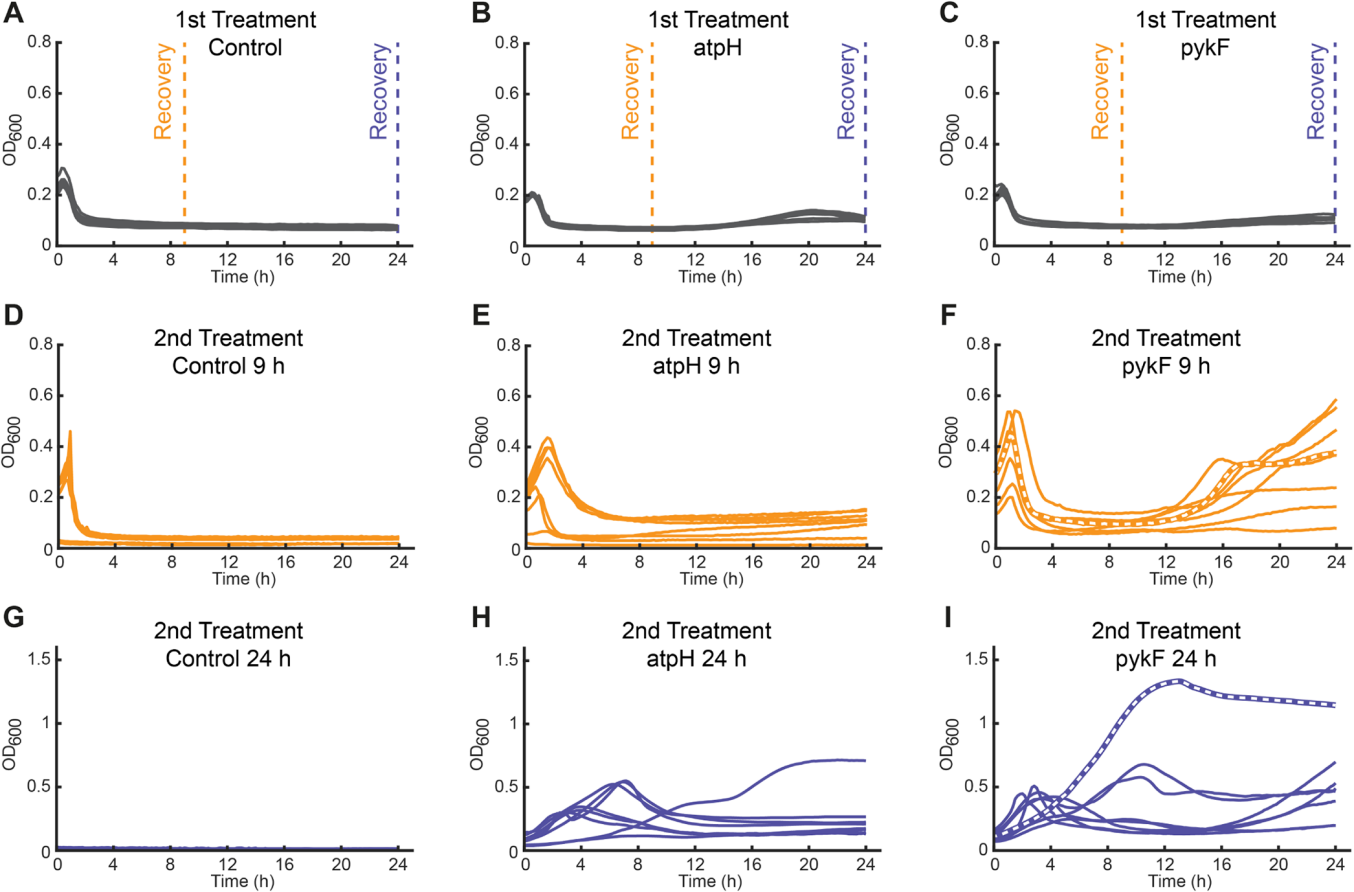
Response of the control strain, the *pykF* strain, and the *atpH* strain to repeated fosfomycin treatment. (A–C) The control strain (A), *atpH* strain (B), and *pykF* strain (C) were treated with 304 μg/mL fosfomycin (*n* = 8). Cells were collected after 9 (orange dashed line) and 24 h (blue dashed line) and recovered in drug-free, rich LB medium for 24 h. (D–F) Cells recovered after 9 h were subjected to the same fosfomycin treatment. (D) 9 h treated control, (E) 9 h treated *atpH,* and (F) 9 h treated *pykF*. (G–I) Cells recovered after 24 h were subjected to the same fosfomycin treatment. (G) 24 h treated control, (H) 24 h treated *atpH,* and (I) 24 h treated *pykF*. Lines in each graph represent different replicates. Thick dashed lines in (F) and (I) indicate the strains used for whole genome sequencing.

To test this hypothesis, we sequenced the genomes of 2 isolates of the *pykF* strain. They were isolated after 9 and 24 h from the cultures that showed the highest fitness after the 24 h treatment (dashed lines in Figure 3). As a reference, we sequenced the original *pykF* strain from the glycerol stock. No mutations were detected in the 9 h isolate compared with the original *pykF* strain. In contrast, the 24 h isolate carried a single mutation that led to an amino acid change (K45I) in *ibaG* (Figure 4). Notably, *ibaG* is located directly upstream of *murA*, and mutations in the *IbaG* homologue *BolA* have been associated with fosfomycin resistance in *Stenotrophomonas maltophilia*.^23^ To determine whether *ibaG* ^K45I^ confers fosfomycin resistance, we introduced this mutation into the ancestral BW25113 strain using a CRISPR method.^24,25^ The resulting strain showed markedly reduced susceptibility to fosfomycin compared to the control strain for gene editing (BW25113 with the plasmids that carry Cas9, sgRNA, and the lambda red system; Figure 4B). The *ibaG* ^K45I^ strain had no growth defect at 2-fold MIC and showed a pronounced regrowth phenotype up to 12× MIC (Figure 4C). The 24 h *pykF* strain showed resistance similar to that of the *ibaG* ^K45I^ strain but had a fitness defect even in the absence of fosfomycin (probably due to the *pykF* knockdown, Figure 4C). These results indicate that the K45I mutation in *ibaG* is the main determinant of the evolved fosfomycin resistance in the isolate of the 24 h *pykF* strain.

**Figure 4:**
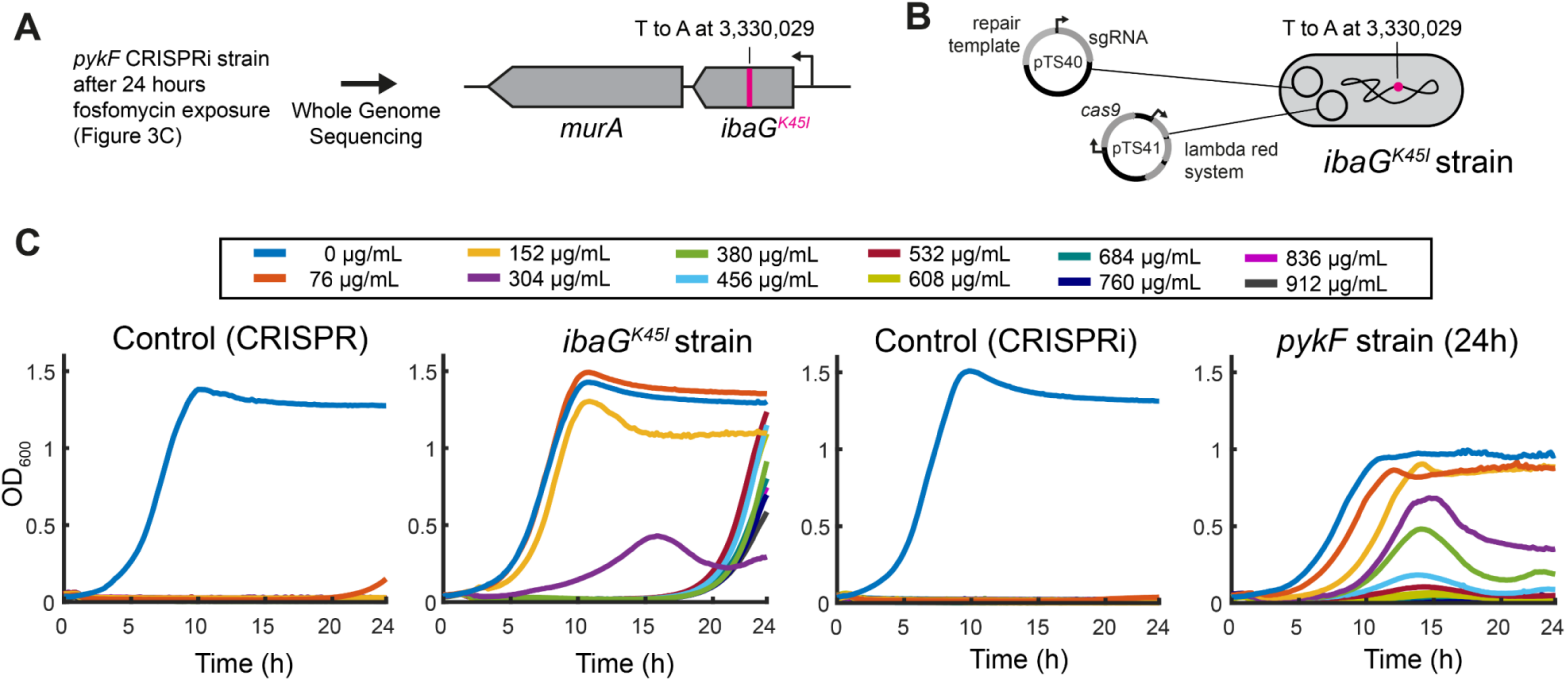
Point mutation in *ibaG* increases resistance to fosfomycin and further enhances *pykF* strain resistance. (A) Whole genome sequencing of *pykF* recovered after 24 h of treatment with fosfomycin identified the *ibaG* ^K45I^ mutation. (B) The *ibaG* ^K45I^ mutation was introduced into *E. coli* BW25113 with a CRISPR method.^24^ (C) *E. coli* BW25113 strain (control with CRISPR plasmids pTS40 and pTS41), BW25113 strain with the IbaG mutation, the control CRISPRi strain, and the *pykF* strain recovered after 24 h of fosfomycin treatment carrying the *ibaG^K45I^* mutation were treated with the indicated concentrations of fosfomycin. All graphs show the mean of *n* = 8 replicates.

### Increases in PEP May Undermine Fosfomycin Activity

Next, we sought to understand the mechanisms by which *pykF* and *atpH* knockdowns reduce susceptibility to fosfomycin. We hypothesized that CRISPRi knockdowns of *pykF* may increase survival rates under fosfomycin exposure by increasing the concentration of the pyruvate kinase substrate PEP. PEP is the cosubstrate of MurA and, at high levels, may compete with fosfomycin for binding to MurA.^26^ To measure PEP, we collected samples for LC–MS/MS analysis from the control strain, the *atpH* strain, and the *pykF* strain before fosfomycin treatment and from cells that had recovered after 9 h of fosfomycin treatment. PEP levels were similar before treatment in all strains (Figure 5A). However, after 9 h of fosfomycin treatment, we observed an increase in PEP levels in the *pykF* strain (4.2-fold), as well as in the *atpH* strain (1.7-fold). The latter had the lowest ATP levels of all three strains, a perturbation that may indirectly lead to higher PEP levels (Figure 5B–D). However, because the control strain did not survive the 9 h treatment, we could not measure its metabolome and can therefore not exclude that high PEP levels are a general response to fosfomycin.

**Figure 5:**
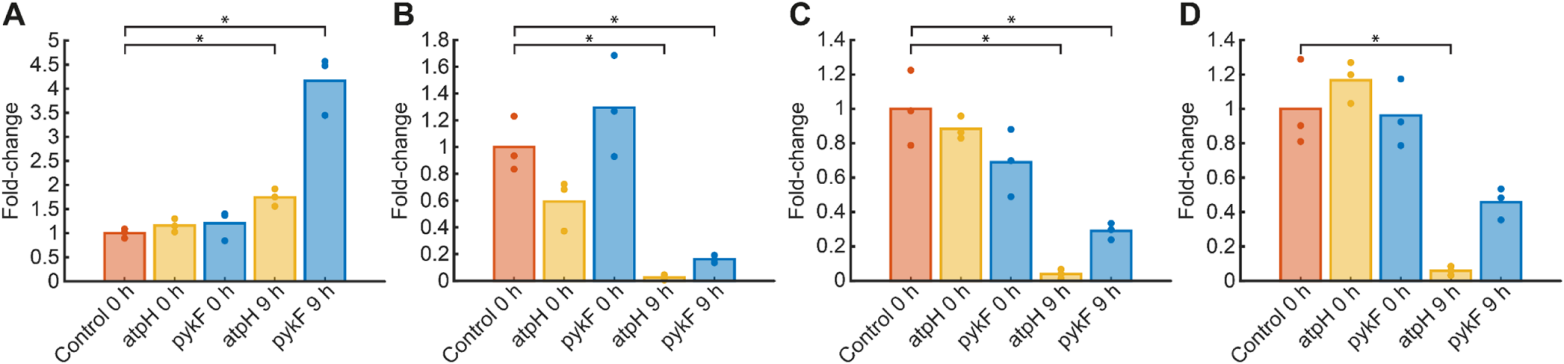
Phosphoenolpyruvate increases in fosfomycin-treated CRISPRi strains. Strains were incubated for 3 h to OD > 0.25 in aTc-containing minimal medium before fosfomycin treatment. Metabolites were measured after 0 and 9 h of fosfomycin treatment (304 μg/mL). Bars represent the mean fold change relative to the control, and dots indicate fold changes of replicates (*n* = 3). Intensities were normalized to the OD. Fold changes relative to the control strain are shown for phosphoenolpyruvate (A), adenosine monophosphate (B), adenosine diphosphate (C), and adenosine triphosphate (D). Statistical significance was determined using one-sided *t* tests against the control strain at 0 h with *p* < 0.05 (*).

### Knockdown of *pykF* and *atpH* Primes the CpxAR Cell Envelope Stress Response System

To gain further insight into the cellular response of fosfomycin-surviving cells, we analyzed the transcriptome of the surviving *pykF* and *atpH* strains after 9 h of treatment. As a reference, we measured the untreated (0 h) control, *pykF,* and *atpH* strains in the absence of fosfomycin (Figure 6). The transcriptome indicated activation of the CpxAR cell envelope stress response system in both the *pykF* and *atpH* strain. The gene that showed the strongest increase after 9 h treatment was *cpxP* (a regulator of *cpxA*). Recent studies have shown that *cpxP* is the most upregulated gene upon activation of the CpxAR system.^27^ Several other genes that are positively regulated by CpxAR were strongly upregulated, including *spy* and *degP*. In contrast, *ompF*, which is negatively regulated by CpxAR, was downregulated. Notably, *cpxP* expression was already increased in untreated cultures (3-fold in *pykF* and 7-fold in *atpH*), suggesting that activation of CpxAR may prime cells to exposure to fosfomycin. A hypothesis is that this basal activation of CpxAR (without fosfomycin) may contribute to heteroresistance by enhancing envelope stress protection in *pykF* and *atpH* strain. Because *cpxP* negatively autoregulates CpxAR activation, this might explain why the growth phenotype is not stable. However, other responses, such as upregulation of *btsT* (encoding the pyruvate:H^+^ symporter) and *metR* (a transcriptional regulator), may also contribute to the regrowth phenotype. Expression of *glpT* and *uhpT*, which are both repressed by CpxAB, remained low across all samples.

**Figure 6:**
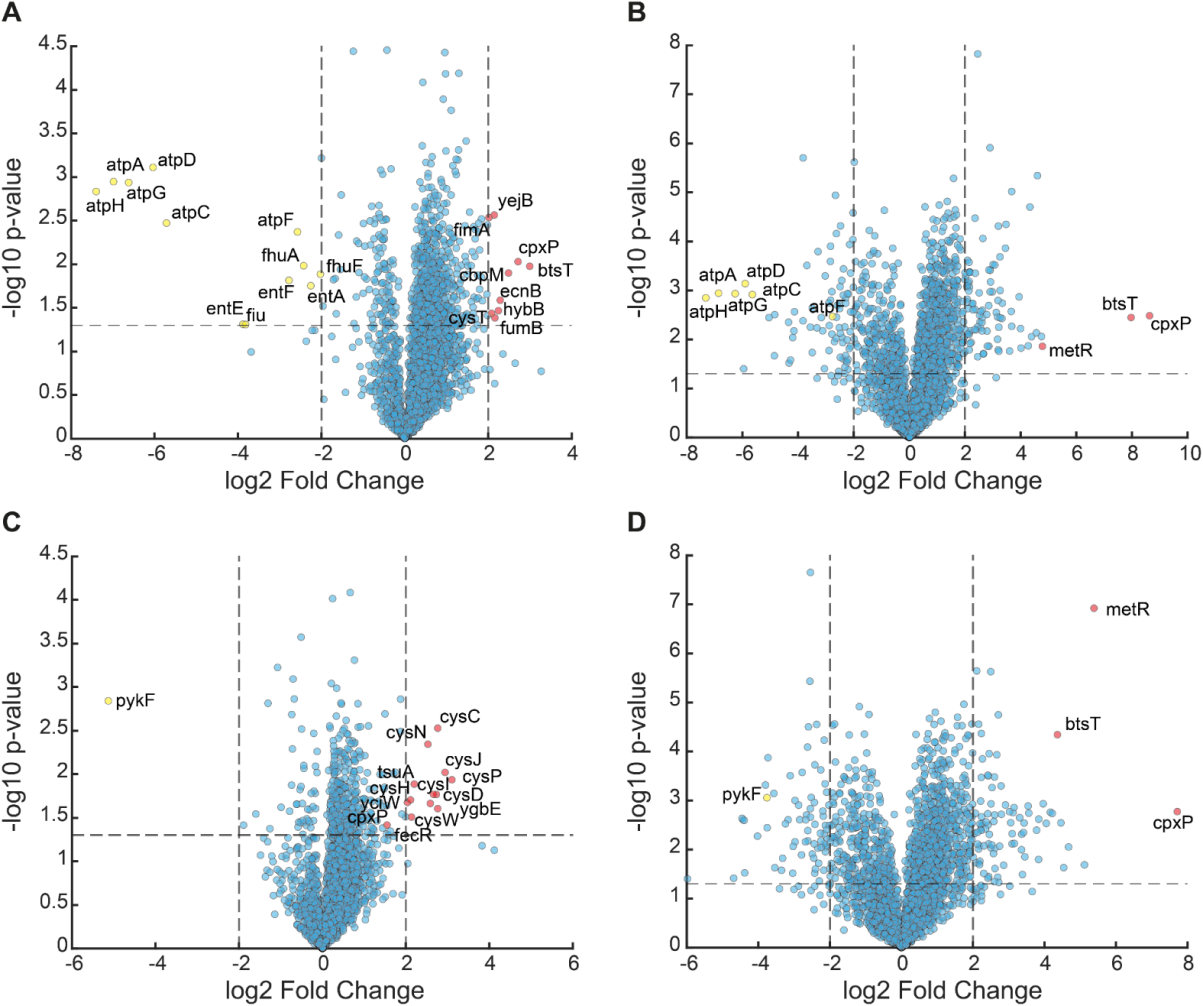
Transcriptome of the *pykF* strain and the *atpH* strain with and without fosfomycin. Strains were incubated for 3 h to OD > 0.25 in aTc-containing minimal medium before fosfomycin treatment. RNA sequencing was performed after 0 and 9 h of fosfomycin treatment (304 μg/mL). Fold changes were calculated relative to the mean of the untreated control strain at *t* = 0 h. (A) Transcript levels of the *atpH* strain before (0 h) and (B) after 9 h of fosfomycin treatment. (C) Transcript levels of the *pykF* strain before (0 h) and (D) after 9 h of fosfomycin treatment.

As expected from the CRISPRi system, transcript levels of the targeted genes were markedly reduced: all ATP synthase genes downstream of *atpH* were significantly decreased in the *atpH* strain (Figure 6A), and *pykF* expression decreased in the *pykF* strain (Figure 6C), thus confirming the efficient repression of the loci targeted by CRISPRi.

## Discussion

Our study reveals that metabolic perturbations can promote the emergence of fosfomycin resistance in *E. coli*. Across our metabolism-wide CRISPRi library, we found that knockdowns of genes encoding pyruvate kinase and ATPase showed a regrowth phenotype during fosfomycin treatment. Notably, this regrowth phenotype occurs also in the control strain but at much lower fosfomycin levels, which is consistent with previous studies.^4^ Population analysis profile tests indicated that regrowth probably results from a heteroresistant subpopulation. The regrowth phenotype was transient because cells that survived 9 h of treatment were killed upon re-exposure to fosfomycin. However, after 24 h of fosfomycin exposure, some cultures acquired stable resistance, and whole-genome sequencing identified a single point mutation, *ibaG* ^K45I^, in an isolate of the *pykF* strain. The *ibaG* gene is located upstream of *murA*, the target of fosfomycin, and therefore, the mutation may change *murA* expression or MurA activity indirectly, but further studies will be required to determine how IbaG mechanistically contributes to resistance. Our metabolite data provided one possible explanation for the transient reduction in fosfomycin susceptibility because in regrowing cells, phosphoenolpyruvate (PEP) levels increased 4-fold in the *pykF* strain and moderately in the *atpH* strain. This supports the hypothesis that PEP accumulation can transiently protect MurA from fosfomycin inhibition. Transcriptome analysis revealed that both *pykF* and *atpH* knockdowns showed strong induction of the CpxAR envelope stress response, and this may also contribute to reduced fosfomycin susceptibility (e.g., by transiently downregulating porins such as OmpF or upregulating periplasmic chaperones). Although we cannot identify the source of heterogeneity, it is not likely that heterogeneity is due to variability in CRISPRi efficiency. This is supported by the strong knockdown of target genes in our transcriptomics data and by previous single-cell studies showing that CRISPRi repression in *E. coli* is uniform across individual cells.^28^ It is possible that multiple phenotypic changes (e.g., CpxAR activation, PEP levels) may result in a fraction of cells that are (temporarily) less susceptible to fosfomycin. This in turn can promote the emergence of stable genetic resistance, such as the *ibaG* ^K45I^ mutation identified here.

We used minimal glucose medium to systematically evaluate how metabolism influences the cellular response to fosfomycin. This becomes difficult in complex media like Mueller–Hinton broth, where nutrients are variable and deplete at different time points during an experiment. Although these controlled conditions differ from an in vivo environment, they allowed us to identify how decreased abundance of pyruvate kinase and ATP synthase can influence responses to fosfomycin. Future studies will need to test whether modulation of pyruvate kinase activity also alters fosfomycin efficacy in vivo, for example, using mouse infection models. Together, our results show how the metabolic state influences fosfomycin efficacy and resistance evolution. Understanding the interplay between metabolism and antibiotic action can inform strategies to prevent resistance evolution, for example, by combining fosfomycin with compounds that increase the activity of pyruvate kinase (e.g., non-PTS sugars like glucose-6-phopshate, which is routinely recommended as a supplement in standard fosfomycin susceptibility tests).

## Methods and Protocols

### Strains

The strains used are shown in Table 1.

**Table 1:** Strains Used

reagent or resource	source	identifier
CRISPRi library in YYdCas9: BW25113 CRISPRipgRNA intC:tetR-dcas9-aadA lacY:ypet-cat	Donati et al., 2021 ^20^	N/A
YYdCas9: BW25113 intC:tetR-dcas9-aadA lacY:ypet-cat (BW25993 *intC:tetR-dcas9-aadA lacY:ypet-cat araB:T7 RNAP-tetA* Δ*araB*)	Lawson et al., 2017 ^38^	N/A
BW25113:ibaG^K45I^	this study	N/A

### Media

Cultivation was performed in LB medium (L3522, Sigma-Aldrich) or M9 minimal medium with 5 g/L glucose as the sole carbon source. M9 medium contained (per liter) 7.52 g Na_2_HPO_4_ 2H_2_O, 5 g KH_2_PO_4_, 1.5 g (NH_4_)_2_SO_4_, 0.5 g NaCl, 10 mL trace salt solution, 2 mL 1.4 mM thiamine-HCl, 1 mL 1 M MgSO_4_, 1 mL 0.1 M CaCl_2_, and 0.6 mL 0.1 M FeCl_3_. The trace salt solution was composed of (per liter) 180 mg CoCl_2_ 6H_2_O, 180 mg ZnSO_4_ 7H_2_O, 120 mg MnSO_4_ H_2_O, and 120 mg CuCl_2_ 2H_2_O. Both media contained 100 μg/mL ampicillin (Amp). For dCas9 expression, anhydrotetracycline (aTc) was added to M9 medium (200 nM). Fosfomycin was added at concentrations of 304 μg/mL unless stated otherwise.

### Screening of Antibiotic Phenotypes of the CRISPRi Library

Using a replicator system (Deutz-System, Kuhner), strains were transferred from flat-bottom 96-well plate glycerol stocks to 96-deep-well plates containing LB medium and incubated for 5 h at 37 °C and 220 rpm. LB culture was diluted in M9 medium in 96-deep-well plates and incubated at 37 °C and 220 rpm for 16 h. Cultures were diluted 150-fold in fresh M9 containing aTc and incubated at 800 rpm and 37 °C in a plate reader (LogPhase 600, Agilent) for 6.5 h. Cultures were 2-fold diluted in M9 containing aTc and 8× MIC fosfomycin (608 μg/mL). Plates were incubated in a plate reader at 800 rpm and 37 °C for 24 h.

### Generation of Growth Curves and Determination of Phenotypes

Growth data were extracted from the plate reader and loaded into MATLAB (R2024b), OD values were converted to OD_600_, and the values were smoothed. Growth data were plotted for all 24 h. Phenotype determination was performed by comparing OD_600_ at time points 0, 4, 12, and 24 h. Each strain had to reach an OD_600_ of at least 0.05 within 24 h to be further considered for phenotypes. Strains were characterized as a tolerant category when tOD_600_ at 4 h was 1.5× higher than OD_600_ at 24 h. The OD increase category required OD_600_ at 12 h to be lower than at 24 h, and resistant category strains had to have increased OD_600_ values over all four time points.

### Regrowth of Fosfomycin-Treated Strains

Strains were inoculated in LB medium and incubated at 220 rpm and 37 °C for 5 h. Subsequently, cultures were diluted in M9 medium in a 96-deep-well plate and incubated at 37 °C and 220 rpm for 16 h. OD_600_ was measured, and cultures were normalized to OD_600_ 0.05 in M9 containing aTc and incubated in a plate reader for ∼6 h at 37 °C, 220 rpm, until OD_600_ of >0.5 was reached. Cultures were diluted 2-fold in flat-bottom plates filled with M9 containing aTc and 8× MIC fosfomycin (608 μg/mL). Plates were incubated in a plate reader at 37 °C and 800 rpm for 24 h. After 9 h, cultures were diluted 200-fold in fresh LB medium, and after 24 h incubation, cultures were diluted again 200-fold in fresh LB medium. LB cultures were incubated for 24 h, and glycerol stocks were created. Glycerol stocks were inoculated in LB medium and incubated at 220 rpm and 37 °C for 5 h. Subsequently, LB cultures were diluted 50-fold in M9 medium in a 96-deep-well plate and incubated at 37 °C and 220 rpm overnight. OD_600_ was measured, and cultures were normalized to OD_600_ 0.05 in M9 containing aTc and incubated in a plate reader for ∼6 h at 37 °C and 800 rpm, until OD_600_ 0.5 was reached for most strains. Then, cultures were diluted 2-fold in two flat-bottom plates filled with M9 containing aTc and 8× MIC fosfomycin (608 μg/mL). Plates were incubated in a plate reader at 37 °C and 800 rpm for 24 h.

### Time-Kill Assay

Strains were inoculated in LB medium and incubated at 220 rpm and 37 °C for 5 h. Subsequently, LB cultures were diluted 100-fold in M9 medium and incubated at 37 °C and 220 rpm for 16 h. OD_600_ was determined, and cultures were started at OD_600_ 0.05 and incubated for ∼3 h at 37 °C and 220 rpm, until OD_600_ 0.25 was reached. Then, fosfomycin (304 μg/mL) was added, and shake flasks were incubated for an additional 24 h at 37 °C and 220 rpm. At time points 0, 0.75, 1.25, 2, 3, and 9 h, aliquots were removed, and a six-instance serial dilution was prepared and spotted on M9 plates containing Amp. After 36 h, pictures were taken, colonies at the dilution with the best resolution were counted, and live cells per milliliter were calculated.

### Population Analysis Protocol Assay

Strains were inoculated in LB medium and incubated at 220 rpm, 37 °C for 5 h. Subsequently, LB cultures were diluted 100-fold in M9 or LB medium and incubated at 37 °C and 220 rpm for 16 h. OD_600_ was determined, and cultures were started at OD_600_ 0.05 in LB or M9 medium containing aTc and incubated at 37 °C and 220 rpm, until OD_600_ > 0.5 (exponential growth) was reached. Cultures were normalized to OD 0.5, an eight-step 10-fold serial dilution series was prepared (10^–1^–10^–8^), and 10 μL dilutions were spotted on M9 and LB agar plates containing aTc and varying concentrations of fosfomycin. Plates were incubated inverted for 36 h at 37 °C, subsequently scanned, colonies counted, and frequencies of resistant cells calculated.

### Metabolomics of *atpH*, *pykF,* and Control Strain

CRISPRi strains were inoculated in LB medium and incubated at 220 rpm and 37 °C for 5 h. Subsequently, cultures were diluted 100-fold in M9 medium in shake flasks and incubated at 37 °C and 220 rpm for 16 h. Cultures were diluted back to a starting OD_600_ of 0.05 in 26 mL M9 medium containing aTc, each in 3 technical replicates and 3 biological replicates in shake flasks. Cultures were incubated for 3 h until OD_600_ 0.25, when 304 μg/mL of fosfomycin was added. Cultures were then incubated at 37 °C and 220 rpm for 9 h. After 6 h, cultures were centrifuged at 2,200 rpm and 4 °C for 5 min, and the supernatants were filtered to reduce cell debris. Cell pellets were resuspended in 25% of the filtered medium and 3 technical replicates were pooled in a fresh prewarmed shake flask to concentrate cells and incubated for 3 more hours. Right before fosfomycin addition and after 9 h treatment, cultures were sampled by centrifugation at 3,150 rpm and 4 °C for 2 min, the supernatant was removed, and pellets were quenched in ice-cold AcN:MeOH:H_2_O (40:40:20). Samples were incubated for 16 h at −20 °C and centrifuged at 17,000 rpm and −9 °C for 5 min, and the supernatants were transferred to fresh reaction tubes and stored at −80 °C until measurement.

Metabolite quantification was carried out using an Agilent 6495 triple quadrupole mass spectrometer (Agilent Technologies), coupled to an Agilent 1290 Infinity II UHPLC system. Metabolite extracts were mixed in a 1:1 ratio with uniformly ^13^C-labeled *E. coli* internal standard prior to analysis. Chromatographic separation was performed with an iHILIC-Fusion­(P) column (50 × 2.1 mm, 5 μm) and an injection volume of 3 μL. Mobile phase A was water with 10 mM ammonium carbonate and 0.2% ammonium hydroxide. Mobile phase B was acetonitrile. The LC gradient was as follows: 0 min, 90% B; 1.3 min, 40% B; 1.5 min, 40% B; 1.7 min, 90% B; 2.0 min, 90% B. Relative quantification of PEP, ATP, ADP, and AMP was performed with an isotope ratio method, and the multiple reaction monitoring (MRM) parameters are given in Table S2. The ratio between the 12C peak height of the sample and the 13C peak height of the 13C internal standard was used for relative quantification.

Statistical analyses were performed in MATLAB (MathWorks, Natick, MA, USA) by the application of the *t* test function.

### Transcriptomics of the *atpH*, *pykF*, and Control Strain

Strains were inoculated in LB medium and incubated at 220 rpm and 37 °C for 5 h. Subsequently, cultures were diluted 100-fold in M9 medium in shake flasks and incubated at 37 °C and 220 rpm overnight. Cultures were normalized to OD_600_ 0.05 and incubated for 3 h in shake flasks at 37 °C and 220 rpm until OD_600_ 0.25 was reached. Two milliliters of each culture were removed and centrifuged for 5 min at 5,330 rpm and 37 °C. The supernatant was removed, and dry pellets were shock-frozen in liquid nitrogen and stored at −80 °C. Fosfomycin was added to a final concentration of 4× MIC (304 μg/mL), and the remaining cultures were incubated for 9 h at 37 °C and 220 rpm. 2× 50 mL per strain were centrifuged at 5,330 rpm and 37 °C for 5 min, supernatant was removed, and dry pellets were shock-frozen in liquid nitrogen and stored at −80 °C. RNA extraction, sequencing library preparation, and NGS sequencing were performed at the Institute for Medical Microbiology and Hygiene (MGM) of the University of Tübingen. Total RNA was quantified using the Qubit RNA Broad Range Assay Kit (Thermo Fisher), and RNA quality was assessed with the Agilent 2100 BioAnalyzer in combination with the RNA 6000 Pico Kit (Agilent Technologies, #5067-1513). RNA was of good quality with a RIN between 6.4 and 9.6. For library preparation, the Illumina Stranded Total RNA Prep Kit was employed, incorporating rRNA depletion with the Ribo-Zero Plus Microbiome kit (Illumina). In brief, 100 ng of total RNA per sample was processed to remove rRNA, followed by the synthesis of cDNA libraries, ligation of adapters, and PCR amplification. The resulting libraries were quantified using the Qubit 1× dsDNA High Sensitivity Assay kit (Thermo Fisher), and the fragment size distribution was analyzed using the High Sensitivity DNA Kit on the Agilent BioAnalyzer. Libraries were then pooled and sequenced on an Illumina MiSeq platform using the MiSeq Reagent Kit v3 (150 cycles), yielding 874,147 to 1,759,004 single-end reads per sample (17,720,891 reads in total). Data processing, including quality control, mapping, and quantification, was done using nf-core/rnaseq v3.18.0 (https://nf-co.re/rnaseq, 10.5281/zenodo.14537300) of the nf-core collection of workflows.^29^ nf-core/rnaseq was executed with Nextflow v24.04.4 ^30^ and Singularity v3.8.7.^31^ The read quality was assessed with FastQC v0.12.1 and led to the removal of around <1% base pairs per sample due to adapter contamination and trimming of low-quality regions with Trim Galore! v0.6.10. rRNA sequences were removed (1.1% to 16% per sample; average: 2.7%) with SortMeRNA v4.3.7.^32^ More than 97% of reads were aligned with STAR v2.6.1d to *E. coli* BW25113 (NCBI RefSeq GCF_000750555.1–RS_2024_06_01) with 4522 genes. Transcripts were quantified by Salmon v1.10.3 ^33^ to transcripts per million (TPM). TPM values were used to calculate log2 fold changes relative to the mean of the control strain. Genes with zero TPM in any sample were excluded. *P*-values were calculated with a two-sided Welch *t* test assuming unequal variances.

### Whole Genome Sequencing

The Core Facility Genomics/NCCT Microbiology (University Hospital Tübingen) performed sample preparation and sequencing. A Qubit dsDNA BR Assay Kit (Thermo Fisher) was used to quantify genomic DNA. Libraries for short-read whole-genome sequencing were prepared using the Illumina DNA Prep (M) Tagmentation kit with 25 ng of DNA and 8 cycles indexing PCR (Illumina DNA/RNA UD Indexes, Tagmentation). Fragment lengths of the library were measured with an Agilent 2100 BioAnalyzer and pooled equimolarly. The pool was sequenced on an Illumina NovaSeq 6000 device (NovaSeq 6000 S4 Rgt Kit v1.5, 300 cycles) with run mode 2× 150 bp paired-end, yielding 3,619,724, 7,215,534, and 5,218,612 paired-end reads for control, 9 h, and 24 h isolates, respectively (230×–450× average sequencing depth per sample). Data processing, including quality control, mapping, and variant calling, was done using nf-core/sarek v3.5.1 (10.5281/zenodo.14886484) of the nf-core collection of workflows.^29,34^ nf-core/sarek was executed with Nextflow v24.04.4 ^30^ and Singularity v3.8.7 ^31^ with default parameters and skipping base recalibration. The read quality was assessed with FastQC v0.12.1, and >99% of reads passed quality filtering by FastP v0.23.4.^35^ Between 88% (24 h) and 96% (control and 9 h) of reads were aligned with BWA-mem v0.7.18 to *E. coli* BW25113 (NCBI RefSeq GCF_000750555.1-RS_2024_06_01). Variants were called with Strelka2 v2.9.10.^36^ Only single nucleotide polymorphisms (SNPs) that passed Strelka2’s quality filter with at least 95% support were considered. Two SNPs were detected by Strelka2 in all three strains compared to the reference BW25113: an A-to-G mutation at positions 360,752 and 1,193,252 in genes *lacI* and *ymfE*, respectively. In the 9 h isolate, no additional SNP was detected, and the 24 h isolate had one additional SNP. Manual inspection with IGV 2.19.6 based on CRAM files showed the 24 h isolate had a 100% T-to-A SNP at BW25113 genome position 3,330,029 in *ibaG* supported by 249 reads.

### Genomic Integration of *ibaG* ^K45I^

The *ibaG* ^K45I^ strain was generated using CRISPR-Cas9 and the λ-Red recombination system.^24,25^ As described previously,^25^ plasmid pT0S41 was transformed into *Escherichia coli* BW25113 by electroporation. Plasmid pTS040 was constructed by assembling the pTS040 backbone with an oligonucleotide that contains sgRNA and the donor DNA that contains the T-A mutation in *ibaG*. After 30 min induction with 7.5 g/L arabinose (λ-Red expression), plasmid pTS040 was transformed by electroporation. The strain was grown for 1 h in SOC medium with kanamycin and 1 μM aTc to induce Cas9 expression and then plated on LB agar with kanamycin, chloramphenicol, and 1 μM aTc. Cells were incubated at 37 °C overnight. The *ibaG* gene was amplified from single colonies by colony PCR. The PCR product was purified (Macherey-Nagel no. 740609), and the mutation was confirmed by sequencing (Microsynth Seqlab).

## Supplementary Material





## Data Availability

Raw sequencing data have been deposited at NCBI in the Sequence Read Archive (SRA) under BioProject accession number PRJNA1290243 (https://www.ncbi.nlm.nih.gov/bioproject/PRJNA1290243).

## Notes

Fosfomycin MIC determination (Figure S1); initial screen growth data of strains added for validation (Figure S2); phenotype validations under fosfomycin treatment (Figure S3); PAP of the control, *atpH*, and *pykF* strain on LB agar and at low fosfomycin concentrations (Figure S4); phenotypes detected in the antibiotic screen; strains that were not found in the initial screen but added as potentially false negatives are marked by dotted lines (Table S1) (PDF)
Source data for all the figures provided in the main text and supporting files (Table S2) (XLSX)
