# Supplementary material for: The Metabolic State of E. coli Influences Fosfomycin Efficacy and Promotes Resistance Evolution
Source: ACS Infect Dis. 2026 Feb 10;12(3):1155–64. doi: 10.1021/acsinfecdis.5c01013 (PMC12993851; doi:10.1021/acsinfecdis.5c01013)
Supplement: Supplementary file 1 [file id5c01013_si_001.pdf]

Supporting Information

The metabolic state of *E. coli* influences fosfomycin efficacy and promotes resistance evolution

Andreas Verhülsdonk[1,2,3], Amelie Stadelmann[1,2,3], Fabian Smollich[1,2,3], Johanna Rapp[1,2,3], Daniel Straub[3,4], Hannes Link[1,2,3,*]

[1]Interfaculty Institute of Microbiology and Infection Medicine, University of Tübingen, 72076 Tübingen, Germany

[2]Cluster of Excellence "Controlling Microbes to Fight Infections", University of Tübingen, 72076 Tübingen, Germany

[3]M3 Research Center, University of Tübingen, Otfried-Müller-Str. 37, 72076 Tübingen, Germany

[4]Quantitative Biology Center (QBiC), University of Tübingen, Otfried-Müller-Str. 37, 72076 Tübingen, Germany

*Corresponding author: hannes.link@uni-tuebingen.de

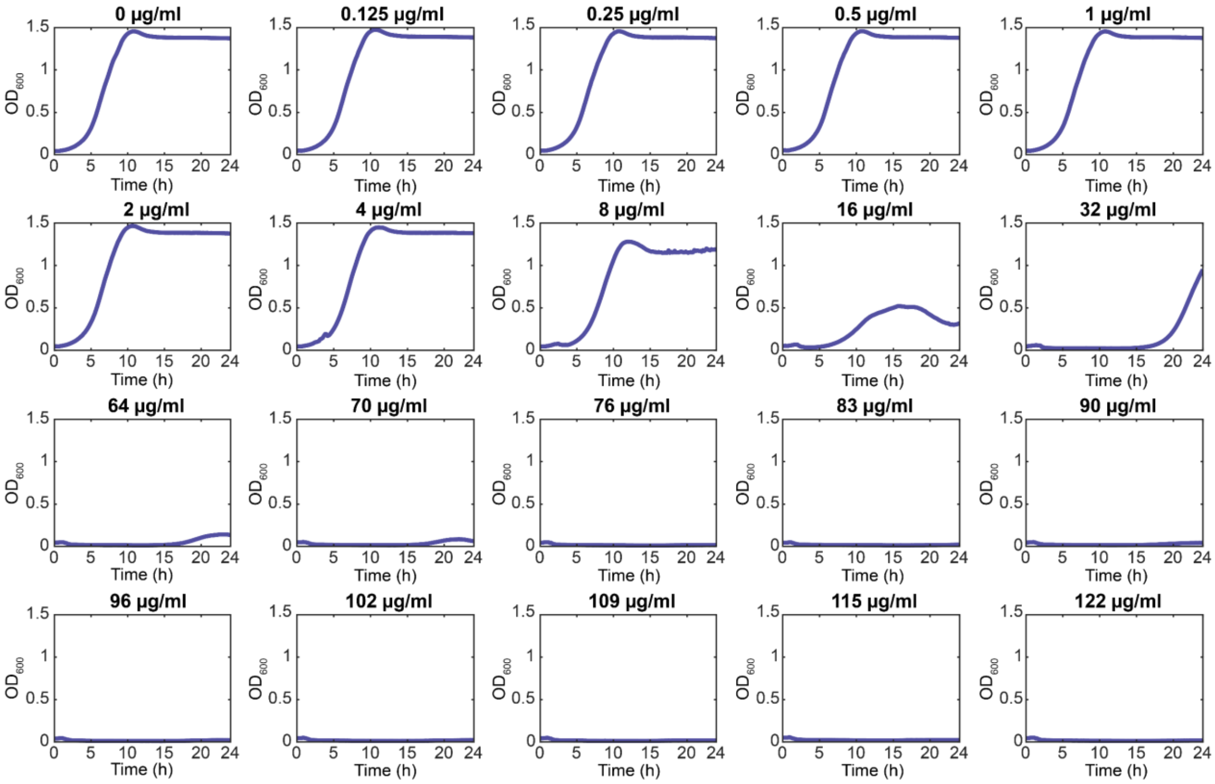

Figure S1. Fosfomycin MIC determination. The control strain was incubated for 24 hours in aTc induced minimal glucose medium under increasing Fosfomycin concentrations (n=8). The first concentration displaying no growth over 24 hours at 76 µg/mL determined the MIC.

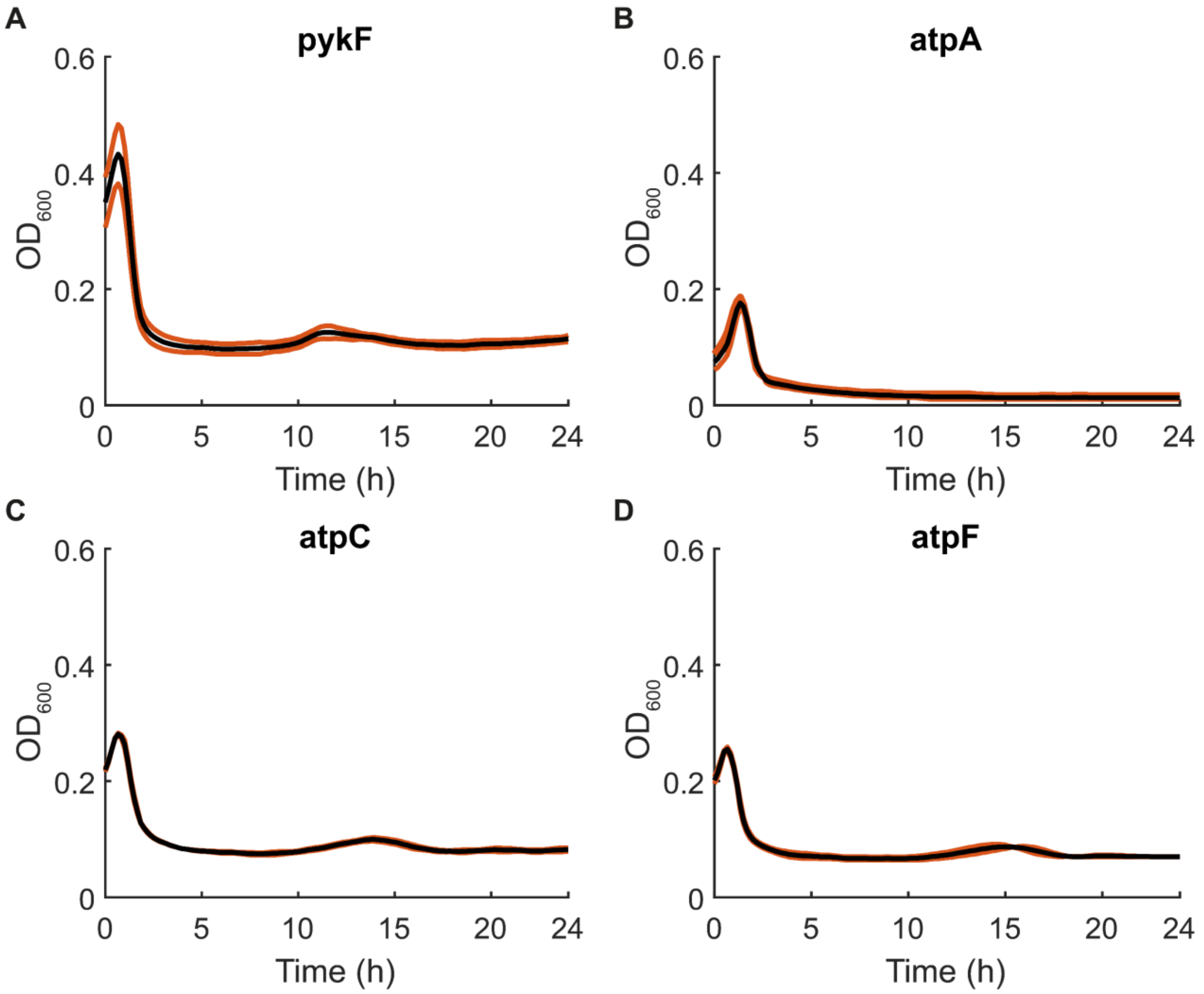

Figure S2. Initial screen growth data of strains added for validation. Strains were incubated for 6.5 hours in aTc induced minimal glucose medium and subsequently for 24 h in aTc induced minimal glucose medium containing 304 µg/mL fosfomycin (n=4). Black lines in each graph indicate mean of all replicates. Colors refer to determined phenotypes in the initial screen: Tolerant (blue), OD increase (orange), and resistant (purple).

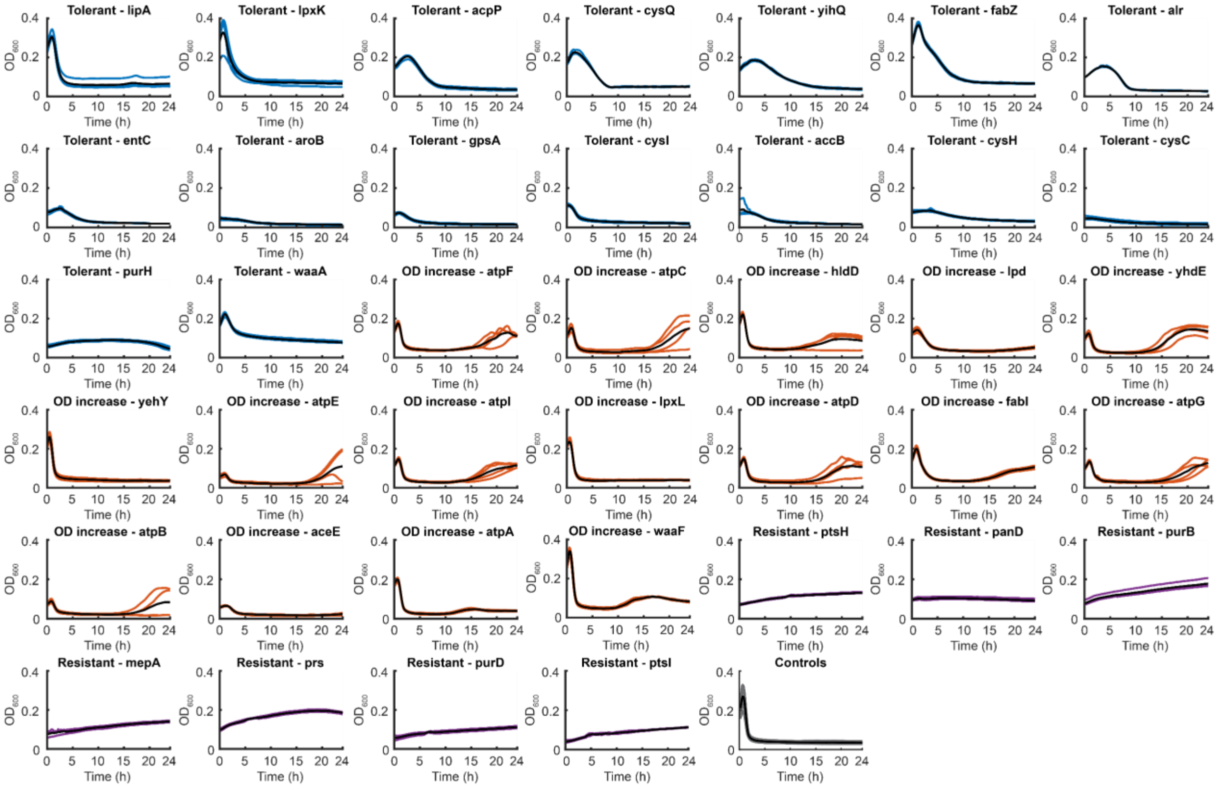

Figure S3. Phenotype validations under fosfomycin treatment. Strains were incubated for 6.5 hours in aTc induced minimal glucose medium and subsequently for 24 h in aTc induced minimal glucose medium containing 304 µg/mL fosfomycin (n=4). Black lines in each graph indicate mean of all replicates. Colors refer to determined phenotypes in the initial screen: Tolerant (blue), OD increase (orange), and resistant (purple).

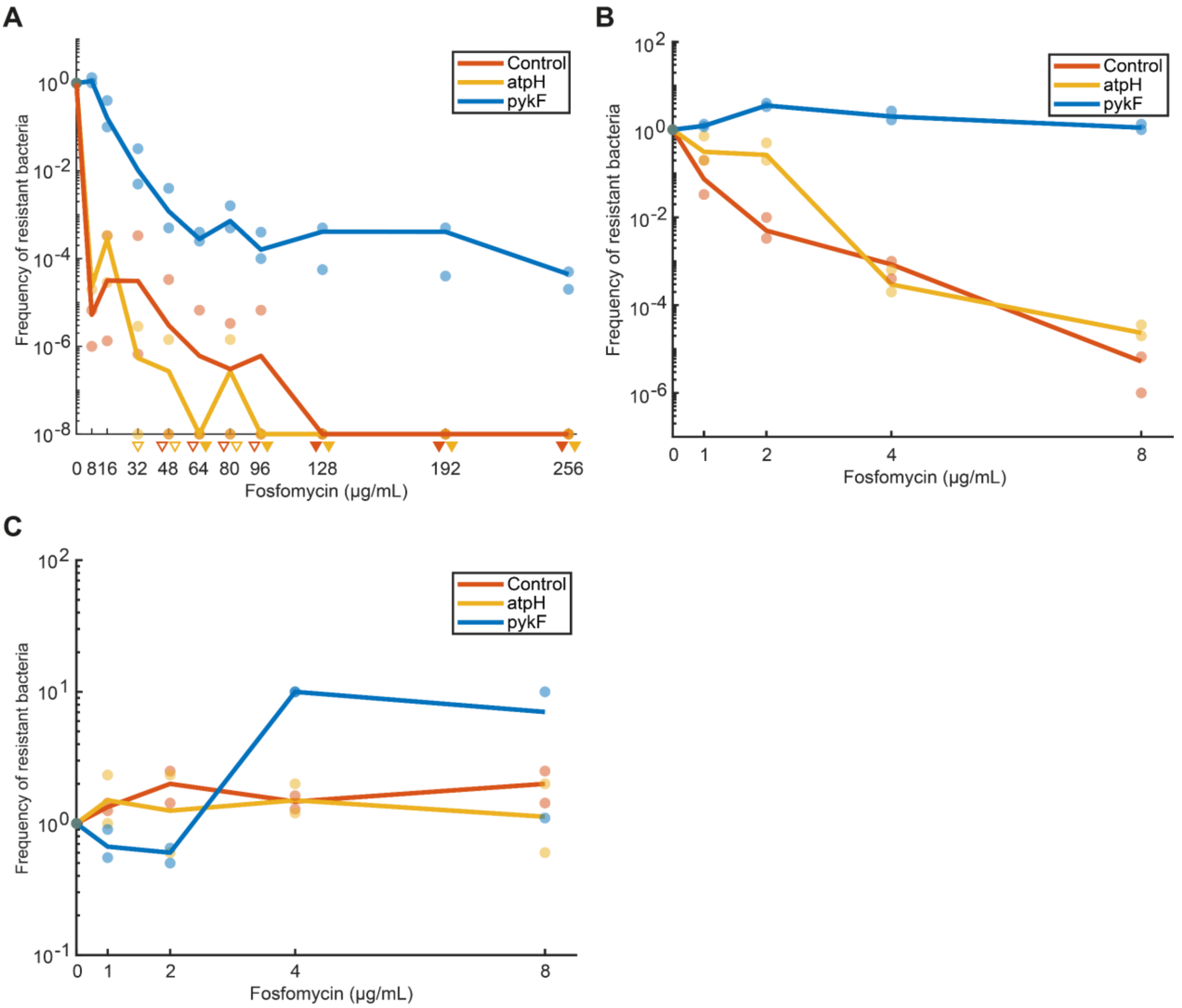

Figure S4. PAP of the control, *atpH*, and *pykF* strain on LB agar and at low Fosfomycin concentrations. Fraction of colony forming units detected in LB medium **(A)** and low concentration fosfomycin in LB medium **(B)** and minimal glucose medium **(C)**. Empty triangles indicate one replicate below the detection limit, filled triangles indicate all replicate below detection limits. Strains were incubated for 24 hours on agar plates containing aTc and increasing concentrations of fosfomycin (n=2).

Table S1. Phenotypes detected in the antibiotic screen. Strains that were not found in the initial screen but added as potentially false negatives are marked by dotted lines.

**Tolerant**	**Resistant**	**OD increase**
accB	mepA	aceE
acpP	panD	atpB
alr	prs	atpD
aroB	ptsH	atpE
cysC	ptsI	atpG
cysH	purB	atpH
cysI	purD	atpI
cysQ		fabI
entC		hldD
fabZ		lpd
gpsA		lpxL
lipA		pykA
lpxK		waaF
purH		yehY
waaA		yhdE
yihQ		pykF
		atpF
		atpA
		atpC
